# Undertaking Research in Other Countries: Further Considerations

**DOI:** 10.1371/journal.pmed.0040187

**Published:** 2007-05-29

**Authors:** Adamson Muula

The article by Skene [1] has touched on an important topic in as far as global health research is concerned. Skene's barometer is certainly a critical contribution to the discourse in research ethics that could be used in both extra-territorial and intra-territorial research. There are, however, several areas where I feel a different opinion would enrich the discussion.

My first concern is that the author presents this barometer with the slices of the pie having sharp demarcations. To the reader, this may suggest that there are clear-cut transitions from one area of the barometer to the other. In reality, however, issues in ethics are less well demarcated. For instance, a research area in itself may fit more in one color zone, but the participants chosen may move it towards the next color zone. Another researcher studying the same research area but different participant groups may be in a different color zone. In general, however, gradations with one color merging into the other, rather than clear-cut demarcations, would be more likely to be observed in practice. The fact that a different scheme could present reality more clearly is exemplified by the author's use of the “green zone”, where research on competent adults, research on vulnerable populations, and research on children have all been grouped under one “roof”. Skene's barometer may also be modified if one considers that vulnerability can be determined on a categorical basis (all persons in that category are vulnerable) versus on a situational basis [2]. For example, why should all persons under sentence of capital punishment be considered vulnerable? Do we assume that these people cannot make informed decisions which are so central in research ethics? Are we worried about coercion or constraining factors?

It is of interest that Skene's barometer has research on stored human tissue and observing people in a public place as neither associated with any laws and no requiring ethics oversight. Did the author mean that a researcher intending to video tape (which is by the way observational) in a restaurant not require ethics oversight? I would argue that stored human specimens should also be associated with ethical oversight. Mfutso-Bengo and I have made a case for continued ethical oversight on stored specimens in international collaborative research [3]. This view has been supported by Ndebele, who has advocated for materials transfer agreements [4]. Although we have made arguments based on actual specimens, we have not argued in support of agreements on use of data that emanates from international research.

The author also writes, “research that imposes severe suffering on animals, especially for a cosmetic rather than scientific purpose, would be widely condemned as well as unlawful in Australia”. I do understand that the author writes from an Australian standard point, but the statement implicitly suggests that research conducted for cosmetic improvements cannot be for “scientific purposes”. What is the author's definition of science? It would certainly make a difference if what the author actually meant was research for cosmetic purposes or gains versus research for treatment of diseases (although cosmetics can also be a treatment for disfiguring human diseases).

It is interesting that the author also suggests that research on cloning “would be unlawful in Australia and almost universally regarded as ethically unacceptable”. This certainly brings into question the thesis that research ethics are universal. I guess in the next decades, the world will grapple with the ethical conduct of research in space. Who has jurisdiction when research occurs in outer space? These questions and others will certainly confront humanity, if not in this century, perhaps in the next.

Finally, because of the use of specific examples and situations, Skene's barometer may be applicable to Australia but not so much to the wider world. I guess the tool will undergo transformations where general algorithms and principles will be considered such that the barometer will be used beyond Australia.

## References

[pmed-0040187-b001] Skene L (2007). Undertaking research in other countries: National ethico-legal barometers and international ethics consensus statements. PLoS Medicine.

[pmed-0040187-b002] Beauchamp TL (2005). How not to rethink research ethics. Am J Bioeth.

[pmed-0040187-b003] Muula AS, Mfutso-Bengo JM (2007). Responsibilities and obligations of using human research specimens transported across national boundaries. J Med Ethics.

[pmed-0040187-b004] Ndebele P (2007). Material transfer agreements are a “must” when transferring specimens across national boundaries. http://jme.bmj.com/cgi/eletters/33/1/35#1312.

